# Fostering improved human islet research: a European perspective

**DOI:** 10.1007/s00125-019-4911-4

**Published:** 2019-06-13

**Authors:** Piero Marchetti, Anke M. Schulte, Lorella Marselli, Eyke Schoniger, Marco Bugliani, Werner Kramer, Lut Overbergh, Susanne Ullrich, Anna L. Gloyn, Mark Ibberson, Guy Rutter, Philippe Froguel, Leif Groop, Mark I. McCarthy, Francesco Dotta, Raphael Scharfmann, Christophe Magnan, Decio L. Eizirik, Chantal Mathieu, Miriam Cnop, Bernard Thorens, Michele Solimena

**Affiliations:** 1 Department of Clinical and Experimental Medicine, Cisanello University Hospital, via Paradisa 2, 56126 Pisa, Italy; 2 Sanofi-Aventis Deutschland GmbH, Diabetes Research, Industriepark Höchst, Frankfurt am Main, Germany; 3 Paul Langerhans Institute Dresden of the Helmholtz Center Munich at University Hospital Carl Gustav Carus and Faculty of Medicine, Dresden, Germany; 4 Clinical and Experimental Endocrinology, University Hospital Gasthuisberg, Leuven, Belgium; 5 Institute for Diabetes Research and Metabolic Diseases of the Helmholtz Center Munich at the Eberhard Karls University of Tübingen, Tübingen, Germany; 6 Oxford Centre for Diabetes Endocrinology and Metabolism, University of Oxford, Oxford, UK; 7 Wellcome Centre for Human Genetics, University of Oxford, Oxford, UK; 8 NIHR Oxford Biomedical Research Centre, Churchill Hospital, Oxford, UK; 9 Vital-IT Group, SIB Swiss Institute of Bioinformatics, Lausanne, Switzerland; 10 Section of Cell Biology and Functional Genomics, Division of Diabetes, Endocrinology and Metabolism, Imperial College, London, UK; 11 Department of Genomics of Common Disease, School of Public Health, Imperial College, London, UK; 12 Department of Clinical Sciences, Faculty of Medicine, Lund University, Malmö, Sweden; 13 Department of Medicine, Surgery and Neuroscience, University of Siena, Siena, Italy; 14 Fondazione Umberto di Mario ONLUS -Toscana Life Sciences, Siena, Italy; 15 INSERM, Cochin Institute, Paris Descartes University, Paris, France; 16 Unité de Biologie Fonctionnelle et Adaptative, Université Paris Diderot, Paris, France; 17 ULB Center for Diabetes Research, Medical Faculty, Université Libre de Bruxelles, Brussels, Belgium; 18 Division of Endocrinology, ULB Erasmus Hospital, Université Libre de Bruxelles, Brussels, Belgium; 19 Centre for Integrative Genomics, University of Lausanne, Lausanne, Switzerland

**Keywords:** Beta cells, Diabetes research, Human islets

*To the Editor*: We read with much interest the review article by Hart and Powers, recently published in *Diabetologia*, on the progress and challenges of the use of human islets in the understanding of islet cell biology and diabetes [1]. In the initial sections of the article, the authors highlight the advances in several areas of human islet cell biology, made possible by the increased availability of islets for research purposes, isolated from the pancreases of organ donors [2, 3]. Such areas include islet architecture, beta cell function and turnover, molecular phenotypes and comparisons with rodent islets. These sections mainly focus on islets from non-diabetic donors, and pay limited attention, if any, to the progress achieved by the use of isolated islets obtained from diabetic individuals. In fact, over the past 10–15 years, several studies have contributed to the identification of islet changes associated with type 1 and, in particular, type 2 diabetes. Although space limitations do not allow a comprehensive listing of all the major advances in this field, we think it is important to summarise at least some key achievements and important differences between ‘type 2 diabetic’ and ‘non-diabetic’ islets (Table 1). They comprise islet morphology and ultrastructure, changes in beta cell identity, insulin secretion defects in response to selective secretagogues (particularly glucose), possible beta cell rescue, mechanisms of islet cell death, the role of genetic and epigenetic factors, gene and protein expression patterns and the search for biomarkers of sick beta cells [4–12]. Taking into consideration the differences between healthy and diseased islet cells is key to elucidating the trajectory of beta cell failure during early glucose intolerance, diabetes onset and disease progression, in order to eventually conceive targeted strategies for the prevention, better treatment and possible remission of this disease.

**Table 1:** Differences in key features of islets isolated from type 2 diabetic vs non-diabetic organ donors

Feature	T2D vs ND islets	Reference
Beta cell identity	Increased number of de-differentiated beta cells, which correlates with the reduction of glucose-stimulated insulin release	[4]
Insulin secretory function	Reduced insulin release in response to acute glucose challenge, associated with lower glucose oxidation Reduced insulin granule exocytosis associated with T2D gene variants	[5, 6]
Beta cell turnover	Increased apoptosis, endoplasmic reticulum stress and islet cell inflammation	[7]
Possible beta cell rescue	Improved insulin secretion from T2D islets after culture	[8]
Epigenetics	Dysregulation of DNA methylation	[9, 10]
Gene expression	Different transcriptome signatures	[11, 12]

ND, non-diabetic; T2D, type 2 diabetic

In the second part of their review, Hart and Powers underline how the characteristics of the islets used in a large proportion of the available studies are inconsistently and marginally reported, making comparisons among studies difficult and scarcely reliable [1]. Hence, the authors propose a list of actions to be put in place, including a record of standardised information on the islets studied, to guarantee more sound and reproducible results. We endorse this request and, certainly, the ongoing discussion will help us to move towards a balance between the need for characterisation and the feasibility of this [13]. Over the past few years, a number of projects on islet pathophysiology have been funded by the European Union, also, in some cases, with the support of the European Federation of Pharmaceutical Industries and Associations (EFPIA), JDRF and charitable trusts (such as the Leona M. and Harry B. Helmsley Charitable Trust). These projects are IMIDIA (Innovative Medicines Initiative for Diabetes: improving beta-cell function and identification of diagnostic biomarkers for treatment monitoring in diabetes, www.imidia.org), T2DSystems (Development of a systems biomedicine approach for risk identification, prevention and treatment of type 2 diabetes, www.t2dsystems.eu), RHAPSODY (Assessing risk and progression of prediabetes and type 2 diabetes to enable disease modification, www.imi-rhapsody.eu) and INNODIA (Translational approaches to disease modifying therapy of type 1 diabetes: An innovative approach towards understanding and arresting Type 1 diabetes, www.innodia.eu). The key participating islet isolating centres have been scrupulously preparing and characterising their human islet preparations (currently more than 400) according to rigorous standardised procedures. The information to be reported on the donors’ clinical characteristics and isolated islet features will be further implemented to comply with the emerging requirements [1, 13]. Importantly, the biorepositories of isolated islets generated in these projects include several well-characterised samples obtained from organ donors with type 2 diabetes, and these are being used to shed further light on the pathophysiology of islet cells in diabetes.

As reported by Hart and Powers [1], the vast majority of studies on human islet cells have employed islets isolated from the pancreas of organ donors. The advantages of this model include the use of transplantation-grade procedures to yield large amounts of islets that can be evaluated in terms of composition, function, survival and molecular properties under different experimental conditions. IMIDIA and RHAPSODY, on the other hand, also introduced the standardised collection and analysis of islet samples obtained following pancreatic surgery from non-diabetic people, individuals with varying degrees of glucose intolerance, and people with recent-onset diabetes or long-standing type 2 diabetes [4]. This has allowed the study of the molecular features of islet cells yielded by laser capture microdissection (LCM) [3, 12], as well as morphometric analysis and study of islet function in fresh tissue slices [14]. One obvious advantage of this approach is that individuals can be metabolically investigated before surgery and, if required, after recovery from the operation. In RHAPSODY, the reliability of this approach has been corroborated by comparing the transcriptome of LCM islets from two cohorts of surgical patients collected at different research sites and according to the same stringent protocols [15] and through the identification of the largest subset of islet expression quantitative trait loci (QTLs) to date [16]. Standardisation of the use of this model in different centres will further contribute to the advancement of human islet research.

## Abbreviations

IMIDIA: Innovative Medicines Initiative for Diabetes: improving beta-cell function and identification of diagnostic biomarkers for treatment monitoring in diabetes
INNODIA: Translational approaches to disease modifying therapy of type 1 diabetes: An innovative approach towards understanding and arresting Type 1 diabetes
LCM: Laser capture microdissection
RHAPSODY: Assessing risk and progression of prediabetes and type 2 diabetes to enable disease modification
T2DSystems: Development of a systems biomedicine approach for risk identification, prevention and treatment of type 2 diabetes
